# Editorial S.I: Early Identification in Autism Spectrum Disorders: The Present and Future, and Advances in Early Identification

**DOI:** 10.1007/s10803-020-04860-2

**Published:** 2021-01-22

**Authors:** Roald A. Øien, Giacomo Vivanti, Diana L. Robins

**Affiliations:** 1 Child Study Center, Yale University School of Medicine, New Haven, CT 06520 USA; 2 Department of Education, UiT—The Arctic University of Norway, Tromsø, Norway; 3 AJ Drexel Autism Institute, Philadelphia, PA USA

**Keywords:** Early identification, Early symptoms, Autism, Phenotype, Screening

## Abstract

Early identification of autism spectrum disorder (ASD) is considered by most scholars and clinicians to be a feasible and useful step for improving the wellbeing of individuals on the autism spectrum and their families. Arguments supporting early detection efforts include the benefit of earlier access to services providing autism-specific evidence-based interventions (Vivanti et al., Journal of Autism and Developmental Disorders, 46(7), 2441–2449, 2016; Zwaigenbaum et al., Pediatrics, 136(Suppl), S10–S40, 2015), and its potential to mitigate or even prevent the challenges associated with ASD symptoms, reduce care costs, and improve the quality of life and productivity of individuals with ASD (Constantino et al., Pediatrics, 146(3), e20193629, 2020; Jacobson et al., Behavioral Interventions, 13(4), 201–226, 1998; Jacobson and Mulick, Journal of Autism and Developmental Disorders, 30(6), 585–593, 2000). Nevertheless, controversies and challenges in this field exist.

## Introduction

Early identification of autism spectrum disorder (ASD) is considered by most scholars and clinicians to be a feasible and useful step for improving the wellbeing of individuals on the autism spectrum and their families. Arguments supporting early detection efforts include the benefit of earlier access to services providing autism-specific evidence-based interventions (Vivanti et al. 2016; Zwaigenbaum et al. 2015), and its potential to mitigate or even prevent the challenges associated with ASD symptoms, reduce care costs, and improve the quality of life and productivity of individuals with ASD (Constantino et al. 2020; Jacobson et al. 1998; Jacobson and Mulick 2000). Nevertheless, controversies and challenges in this field exist. These include concerns related to the potential harms of classification errors, the potential burden of universal screening on chronically under-resourced service systems (Hickey et al. 2020), the limited knowledge on the long-term outcomes of children detected and treated early (Siu et al. 2016), and the variable performance of existing tools used for early identification across individuals and contexts.

To address these gaps and controversies, a critical challenge in our field is the establishment of a solid evidence base concerning (1) the outcomes of children detected in the early developmental period, (2) the tools and approaches that facilitate accurate and feasible identification, and (3) the factors that act as barriers and facilitators to successful implementation of early detection procedures, including both societal and individual variables. The current special issue provides critical knowledge across each of these areas.

### Early Manifestations and Outcomes of Children Identified Earlier

This special issue includes four studies (Hatch et al. 2020; Lorenzo et al. 2020; Stenberg et al. 2020; Tanner and Dounavi 2020) that provide new insight on early emerging symptoms. This is a critical area of investigation because the age of diagnosis in epidemiological samples is still 3–5 years of age (Baio et al. 2018; Fountain et al. 2011; Mandell et al. 2010; Shaw et al. 2020). Previous studies have found a gap between the onset of parental concern, which have been found to emerge at a median age of 15-months (Chawarska et al. 2006) and the age of diagnosis, highlighting the difficulties of early identification of the broader autism spectrum. More knowledge about both the children who are identified and those who were missed early in the developmental period is of great importance for early identification, thus improving outcomes and quality of life. The discrepancy between the age of diagnosis, parental concern, and knowledge of diagnosis’s stability has multiple causal factors, including heterogeneity in etiology, behaviors, core symptoms, cognitive skills, adaptive skills, language, and communication, the onset of diagnosis, and core symptom in ASD (Zwaigenbaum et al. 2015).

This special issue includes studies that address various manifestations of the disorder in the early developmental period. For example, Hatch et al. (2020) presents findings related to diminished response to name. Response to name is a known potential marker of the disorder, and is also evaluated in diagnostic measures, such as the Autism Diagnostic Observation Schedule, 2nd Edition (ADOS; (Lord et al. 1999). The study examine the potential of response to name as a potential marker for both ASD and Attention-Deficit/Hyperactivity Disorder (ADHD). Hatch and colleagues examined children at various timepoints between 6 and 36 months of age and found impaired response to name to be a general marker for ASD or ADHD early in the developmental period, but specific to ASD in older toddlers. Lorenzo et al. (2020) investigated another potential early marker for ASD, examining differences between 10-month old toddlers processing of spatial frequencies and discrimination between fearful and neutral facial expressions. Stenberg et al. (2020) assessed cognitive, functional language and symptom severity in children that were invited to participate in the Norwegian Mother, Father and Child (MoBa) study’s sub-study: the Autism Birth Cohort (ABC) study. Children were invited based upon various markers for concern raised by parents or professionals at 18 and 36 months and assessed at 42 months of age or later. This study highlights differences in sub-groups of children with concern and case–controls in terms of cognitive abilities, functional language, and symptom severity. Lastly, this special issue includes a systematic literature review of studies from the last 6 years that have investigated symptoms related to ASD before 18 months of age. Tanner and colleagues (2020) included studies that reported symptoms prior to 18 months in children who later received an ASD diagnosis. To summarize, efforts in the field are valuable in identifying markers for early identification of the disorder, both in those who are identified early by early identification efforts, and for those who are identified later in childhood. This is of importance as some might not have evident symptoms at 18 or 24-months of age, and thus receive a diagnosis later in childhood. More knowledge of early symptoms in different sub-groups of children identified and diagnosed at various timepoints are important to secure more children on the spectrum early interventions, and better long-term outcomes (Vivanti et al. 2016).

### Approaches to Early Detection

Eight articles in this SI (Lai et al. 2011; Lorenzo et al. 2020; Mackie et al. 2020; Meagher et al. 2020; Parikh et al. 2020; Sacrey et al. 2020; Wu et al. 2020) provide new important information on tools and procedures to facilitate early detection. The majority of these papers described early detection strategies that relied on parent report, but varied in recruitment of participants (high-risk siblings of children with ASD, children receiving early intervention) and emphasis on standardized screening versus parent contributions. When parent concerns were elicited in addition to standardized screening (Pasco et al. 2019), high-risk siblings who were later diagnosed with ASD had higher screening scores and also more concerns raised by parents at 14 months. A striking difference with prior literature was seen in the type of concerns parents reported; unlike studies both with high-risk siblings (Ozonoff et al. 2009) and children identified from primary care screening (Richards et al. 2016), disruptive behaviors were most commonly reported, rather than language concerns. This may explain the finding that parent concern related to outcomes of ASD or other developmental disorders, rather than being specific to ASD. Sacrey et al. (2020) collected standardized parent report on high-risk siblings and low-risk children for comparison; parent report at 9 months was highly predictive of autism diagnosis at age 3 years. Another study involving high-risk siblings (Parikh et al. 2020) demonstrated that the specificity of parent-report screening improves from 6 to 36 months. The authors emphasize that this should not lead to recommendations against very early screening, given the importance of diagnosis to inform ASD-specific early intervention, similar to other papers that compare properties of screening tools when utilized at different ages (Dai et al. 2020; Sturner et al. 2017).

Two papers reported on different aspects of screening children enrolled in early intervention (Eisenhower et al. 2020; Mackie et al. 2020). The first examined the engagement of parents and early intervention providers in a two-stage screening process, culminating in ASD evaluations for at-risk children. They found high levels of participation from families with children of color and those on public insurance, suggesting that health disparities in ASD diagnosis can be reduced by screening children after enrollment in early intervention. The second focused on parents’ engagement in the screening process. Parents increased their understanding of symptoms, prognosis, and treatment, as well as differentiation of ASD from other developmental disorders as they progressed through the screening and evaluation process, which sheds light on important aspects to discuss with parents who may be reluctant to consider ASD risk at initial screening.

One paper focused on a more cost- and time-effective alternative to a formal diagnostic tool (Wu et al. 2020); using the Screening Tool for Autism in Toddlers (Stone and Ousley 2004), investigators classified children with ASD and developmental delay (DD) with accuracy nearly as high as the Autism Diagnostic Observation Schedule (Lord et al. 1999). Finally, one study described an alternative approach to detecting ASD risk in young children. Meagher and colleagues (2020) reported that challenging behavior during auditory evaluation increased risk of later ASD diagnosis by more than fivefold. The moderately low sensitivity (56.3%) indicates that ASD cannot be ruled out by absence of challenging behavior during exam; however, high specificity (81.4%) suggested that children who had difficulty tolerating the audiology exam should be referred for ASD evaluation, given that more than half of the children later diagnosed with ASD would be identified by exhibiting interfering behavior.

Taken together, these papers emphasize the value of behavioral indicators of ASD risk in young children, however there are multiple factors that affect the process of early identification, which is also included in this special issue.

## Factors that Affect Early Detection Efforts

Finally, several studies in our special issue examined factors that contribute to variability in the performance of screening tools and implementation outcomes when early detection procedures are used in specific populations and integrated in specific health services (Christopher et al. 2020; Iskrov et al. 2019; Koller et al. 2019) This is a critical yet understudied area in the literature, and the resulting gap in knowledge results in limited guidance on how to ensure successful efforts to implement early screening procedures across populations and contexts. The discrepancy between philosophical adherence to the principle that early detection strategies are important for early diagnosis and intervention, and the rigorous implementation of such strategies is a likely contributor to delays in diagnosis. For example, the study by Iskrov et al. (2019) documents how a majority of surveyed pediatricians and child psychiatrists in Bulgaria support the idea of a national program for ASD early screening—however, this aspiration is curbed by limited synchronization and standardization of early detection practices in the country. Importantly, a lack of standardization in screening procedures has been observed even when screening tools have clear administration guidelines. For example, there is evidence that M-CHAT is often not administered as intended by healthcare professionals, with deviations from the protocol ranging from selective administration to specific children, selective referrals of children who screen positive, and incomplete administration (García et al. 2019; Monteiro et al. 2019; Øien et al. 2019; Sánchez-García et al. 2020), while others have not conducted the follow-up routine of the screen positives (Stenberg et al. 2014). Although lack of adherence to prescribed protocols likely compromises the utility of screening tools, little attention has been devoted to fidelity of implementation in the area of autism early detection. The introduction of fidelity monitoring procedures has been a critical step for improving the success of population-based screening protocols for alcohol consumption (Williams et al. 2015) and obesity risk (Aveyard et al. 2016) in primary care settings, sexual health programs (MacDonald et al. 2016) and other areas of healthcare (Tucker and Blythe 2008). In the field of autism there is an increasing emphasis on the role of fidelity of intervention procedures for the success of treatment programs (e.g., Verschuur et al. 2019). The same focus should be extended to implementation fidelity of early screening procedures.

Another factor contributing to different rates of children being detected and referred to diagnostic evaluations at an early age is sociodemographic characteristics. As shown by the article of Koller et al. (2019) subtle but revealing differences exist in the characteristics and timing of detection and diagnosis among different ethnic groups in Israel—a finding consistent with data from other countries (Daniels and Mandell 2014). Finally, as shown by the articles of Christopher et al. (2020), child characteristics other than core ASD symptoms appear to affect the result of screening tools, including emotional and behavior problems.

Summarizing, this special issue of JADD provides the readers with new knowledge deepening our understanding of behavioral approaches to detection of ASD risk in young children, as well as highlighting novel applications of technology to identify risk. A recurring theme across the articles in the special issue is the interplay between child factors (including early manifestations of ASD risk, other condition, their behavior during check-ups, and demographics) and factors related to healthcare systems and screening procedures in contributing to the success of early identification efforts. Further, the papers in this issue provide actionable insight on which strategies work and for whom, while also highlighting that there is still much work to be done to decrease the age of diagnosis across implementation contexts and populations. Many of the papers in this SI focus on a specific country or region; however, their conclusions likely will generalize to other parts of the world, although cross-validation in different cultures is critical to ensure strategies produce comparable results (Brickman et al. 2006) for a discussion of cross-cultural issues in neuropsychological testing, more broadly, and de Leeuw et al. (2020) for a framework for cultural factors in autism identification and diagnosis (de Leeuw et al. 2020). It is clear from the geographic representation of the studies included in the special issue (Bulgaria, Canada, Netherlands, Norway, Israel, US, UK, Taiwan) that early detection of autism is of global importance, and further studies should take culture and ethnicity into consideration when examining early markers and symptoms of ASD.

With respect and best regards,

The editors: Dr. Roald A. Øien, Ph.D. (Professor UiT—The Arctic University of Norway), Dr. Giacomo Vivanti (Associate Professor—Drexel University), and Dr. Diana L. Robins, Ph.D. (Professor and Director of the A. J. Drexel Autism Institute—Drexel University).
